# Particulate matter air pollution disrupts endothelial cell barrier via calpain-mediated tight junction protein degradation

**DOI:** 10.1186/1743-8977-9-35

**Published:** 2012-08-29

**Authors:** Ting Wang, Lichun Wang, Liliana Moreno-Vinasco, Gabriel D Lang, Jessica H Siegler, Biji Mathew, Peter V Usatyuk, Jonathan M Samet, Alison S Geyh, Patrick N Breysse, Viswanathan Natarajan, Joe G N Garcia

**Affiliations:** 1 Institute for Personalized Respiratory Medicine, Section of Pulmonary, Critical Care, Sleep and Allergy, Department of Medicine, University of Illinois at Chicago, Chicago, IL, USA; 2 Department of Preventive Medicine, Keck School of Medicine, University of Southern California, Los Angeles, CA, USA; 3 Department of Environmental Health Science, Bloomberg School of Public Health, Johns Hopkins University, Baltimore, MD, USA

**Keywords:** Calpain, Endothelial permeability, Particulate matter, ROS, TRPM2

## Abstract

**Background:**

Exposure to particulate matter (PM) is a significant risk factor for increased cardiopulmonary morbidity and mortality. The mechanism of PM-mediated pathophysiology remains unknown. However, PM is proinflammatory to the endothelium and increases vascular permeability *in vitro* and *in vivo* via ROS generation.

**Objectives:**

We explored the role of tight junction proteins as targets for PM-induced loss of lung endothelial cell (EC) barrier integrity and enhanced cardiopulmonary dysfunction.

**Methods:**

Changes in human lung EC monolayer permeability were assessed by Transendothelial Electrical Resistance (TER) in response to PM challenge (collected from Ft. McHenry Tunnel, Baltimore, MD, particle size >0.1 μm). Biochemical assessment of ROS generation and Ca^2+^ mobilization were also measured.

**Results:**

PM exposure induced tight junction protein Zona occludens-1 (ZO-1) relocation from the cell periphery, which was accompanied by significant reductions in ZO-1 protein levels but not in adherens junction proteins (VE-cadherin and β-catenin). N-acetyl-cysteine (NAC, 5 mM) reduced PM-induced ROS generation in ECs, which further prevented TER decreases and atteneuated ZO-1 degradation. PM also mediated intracellular calcium mobilization via the transient receptor potential cation channel M2 (TRPM2), in a ROS-dependent manner with subsequent activation of the Ca^2+^-dependent protease calpain. PM-activated calpain is responsible for ZO-1 degradation and EC barrier disruption. Overexpression of ZO-1 attenuated PM-induced endothelial barrier disruption and vascular hyperpermeability *in vivo* and *in vitro*.

**Conclusions:**

These results demonstrate that PM induces marked increases in vascular permeability via ROS-mediated calcium leakage via activated TRPM2, and via ZO-1 degradation by activated calpain. These findings support a novel mechanism for PM-induced lung damage and adverse cardiovascular outcomes.

## Background

Ambient particulate matter (PM) poses a threat to national public health in urban environments and other polluted areas throughout the US and around the world. Epidemiological studies have shown associations of exposure to low levels of urban particulate matter with increased cardiopulmonary morbidity and mortality [1,2]. The assessment of PM-induced health effects is challenging. Various mechanisms have been proposed to explain the cardiopulmonary health effects of PM including increased pulmonary and systemic oxidative stress and inflammation, enhanced coagulation, and altered cardiac autonomic function [3,4].

Airway epithelium represents a well-investigated target for environmental pollutants such as PM. Exposure of airway epithelium to airborne PM causes altered cytokine/chemokine gene expression and increased production of IL-1β, IL-6, IL-8 and TNF-α [5,6]. Now, the lung endothelium is also gaining attention as a viable PM target tissue. Exposure of the endothelium to PM or its active components in the systemic circulation induces significant systemic endothelial inflammation and dysfunction, even at low levels of exposure [7,8]. The water soluble fraction of PM (up to 35-50%) can easily diffuse through the epithelium/endothelial barrier to the systemic circulation. Bioavailable transition metals present in urban PM catalyze redox reactions in human lung endothelium, which cause oxidative stress, increase the production of inflammatory cytokines, and increase the activation of NF-κB signaling pathways, all of which trigger further endothelial damage [9]. Increased endothelial monolayer permeability is also observed in inflammatory pulmonary conditions such as acute lung injury (ALI), acute respiratory distress syndrome (ARDS), and sepsis; devastating lung disorders with mortality exceeding 30%, as well as in more subacute and chronic inflammatory disorders such as asthma [10,11].

We recently described a murine asthma model with strong evidence for PM-mediated vascular barrier dysfunction with increased protein leakage into bronchoalveolar lavage (BAL), a marker of acute inflammatory lung damage [12]. By assessment of direct effects on endothelial barrier integrity *in vitro*, we further demonstrated that the vascular hyperpermeability mediated by intratracheal PM exposure is mainly dependent on acute endothelial barrier disruption by PM [13]. Exposure of human lung EC to PM resulted in significant ROS generation, which mediates p38beta MAPK activation, leading to the phosphorylation of HSP27 [13]. Phosphorylated HSP27 facilitates the synthesis of stress fibers and formation of paracellular gaps, which causes protein-containing fluid to leak from the microvessel lumen to the lung alveoli, leading to further pulmonary inflammation [13]. However, these previous findings did not explain the persistent character of PM-mediated endothelial barrier disruption.

To fully examine PM-mediated vascular hyperpermeability, we explored effector targets such as tight junction and adherens junction proteins, which are known to be critical in EC barrier maintenance. We demonstrated that PM exposure induced the relocation of tight junction protein Zona occludens-1 (ZO-1) from the cell periphery, which was accompanied by a significant reduction in the level of ZO-1 protein but not in the levels of adherens junction proteins (VE-cadherin and β-catenin). PM also mediated intracellular calcium mobilization via the transient receptor potential cation channel M2 (TRPM2), in a ROS-dependent manner with subsequent activation of the Ca^2+^-dependent protease calpain. PM-activated calpain is responsible for ZO-1 degradation and EC barrier disruption. These observations not only provide new information as to how PM disrupts endothelial tight junctions, but also represent the first evidence establishing the critical role of calpain signaling in modulating endothelial cell barrier function under oxidative stress. These results increase our understanding of PM-induced adverse cardiopulmonary outcomes. Moreover, as the newly characterized signaling cascade of ROS/TRPM2/Calpain/ZO-1 likely has fundamental roles in regulating the cytoskeleton under oxidative stress, these novel observations may have broad applicability to vascular pathophysiology in a variety of cell types.

## Methods

### Reagents and chemicals

Molecular mass standards, polyacrylamide gels, and protein assay reagents were purchased from Bio-Rad (Hercules, CA). A number of antibodies were purchased including ZO-1 (BD, Franklin Lakes, NJ), ZO-2, VE-cadherin, β-catenin (Santa Cruz, Santa Cruz, CA), and TRPM2 (Bethyl Labs, Montgomery, TX). Endotoxin ELISA kit was purchased from Uscn Life Science (Wuhan, China). TRPM2 siRNA was obtained from Dharmacon (Lafayette, CO) and all other chemicals and reagents were obtained from Sigma-Aldrich (St. Louis, MO) unless stated otherwise.

### PM

PM sample (0.1-0.3 μm of aerodynamic diameter) was collected (April of 2005) from the Ft. McHenry Tunnel, Baltimore, MD using a high-volume cyclone collector [6,12-15]. The elemental composition (micrograms per gram) of this PM sample representing the most abundant constituents included: Fe 57 (132,545.9), Ca 43 (47,263.08), Al 27 (17,533.74), Cu 63 (8,777.886), Na 23 (8,068.263), Mg 25 (7,332.056), S 34 (6122.67), K 39 (5,102.741), Ti 47 (3,197.332), Mn 55 (2133.87), Zn 66 (645.60), Cr 52 (325.97), Pb 208 (122.07), Pb 204 (119.71), Sr 86 (111.01), Sr 88 (110.79), V 51 (76.30), Ni 60 (59.61), La 139 (21.27), Sn 118 (20.84), Sb 121 (15.76). No detectable endotoxin contamination was found in the PM suspension (1 mg/ml) in water by LPS ELISA kit (Uscn Life Science).

### Human endothelial cell culture

Human lung microvascular ECs obtained from Lonza (Basel, Switzerland) were cultured as previously described [16] in EGMMV-2 complete medium (Lonza).

### Transendothelial electrical resistance (TER)

Endothelial cells were grown to confluence in polycarbonate plates containing evaporated gold microelectrodes, and TER measurements were continuously obtained using an electrical cell-substrate impedance sensing system (ECIS) (Applied Biophysics, Troy, NY) as previously described in detail [17].

### siRNA transfection of endothelial cells

Human microvascular lung ECs were transfected with siRNA using siPORT Amine (Ambion, Austin, TX) according to the manufacturer's protocol as we described previously [13].

### Measurement of intracellular Ca^2+^

ECs plated on glass cover slips were loaded with 5 μM fura-2 AM (Invitrogen, Carlsbad, CA) in 1 ml of basal medium as previously reported [18]. The cover slips with ECs were inserted diagonally into 1 cm acrylic cuvettes filled with 3 ml of basal medium at 37°C. Fura-2 fluorescence was measured with an Aminco-Bowman Series 2 luminescence spectrometer (SLM/Aminco, Urbana, IL) at excitation wavelengths of 340 and 380 nm and emission wavelength of 510 nm. The cover slips with ECs were then moved to 35 mm dishes, treated with PM suspension, and incubated for 15 minutes at 37°C in 95% O_2_ and 5% CO_2_. After every 15-minute incubation, the coverslips were withdrawn from the dish and inserted back into the acrylic cuvettes for Fura-2 fluorescence measurement, then returned to the dish with the resuspended PM preparation for another incubation period (15–60 min).

### Animals

Male A/J mice (10–12 weeks of age; Jackson Laboratories, Bar Harbor, ME) were housed in an environmentally controlled animal facility at the University of Illinois at Chicago (UIC) for the duration of the experiments. All animal procedures follow the guideline of the UIC Animal Care and Use Committee. PM (10 mg/kg, in 50 μl of saline) was delivered via intratracheal aspiration 1 hr after NAC or calpeptin treatment, as previously described [12,15]. Animals were sacrificed 24 hr after PM treatment, and bronchoalveolar lavage (BAL) and lung tissue were collected [12,19]. Total BAL cells were counted with a hemocytometer. The BAL fluid was used for protein and cytokine measurement (Bio-Rad, Hercules, CA) according to the user’s manual.

### Statistical analysis

Data are presented as group means ± SEM. We performed statistical comparisons among treatment groups by randomized-design two-way analysis of variance followed by the Newman-Keuls post hoc test for more than two groups, or by an unpaired Student’s *t*-test for two groups. In all cases, we defined statistical significance as p < 0.05.

## Results

### PM induces endothelial barrier disruption and tight junction protein degradation

**Figure 1:**
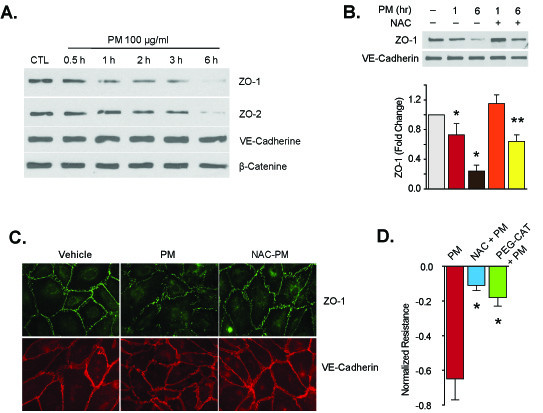
**PM induces ROS-dependent EC barrier disruption and ZO-1 degradation. (A)** Human lung microvascular ECs grown on 6-well plates were challenged with PM (100 μg/ml) for 0.5-6 hr. Cell lysates were analyzed by Western blotting with antibodies to ZO-1, ZO-2, VE-cadherin, and β-catenin. **(B)** Human lung microvascular ECs grown on 6-well plates were challenged with PM (100 μg/ml) for 1 or 6 hr with or without NAC (5 mM, 1 hr pretreatment). Cell lysates were analyzed by Western blotting. Changes in levels of ZO-1 are expressed as fold changes and normalized to control. Shown are representative blots from three independent experiments. Relative levels of ZO-1 densitometry are shown in bar graph under corresponding experimental conditions. N = 3. *p < 0.05 compared to control. **p < 0.05 compared to PM-6 hr group. **(C)** Human lung microvascular ECs grown on glass chamber slides were challenged with PM (100 μg/ml) for 6 h with or without NAC (5 mM, 1 hr pretreatment). VE-cadherin and ZO-1 localization was detected by immunofluorescence staining with antibodies to VE-cadherin and ZO-1. **(D)** ECs grown on ECIS gold electrodes were pretreated with NAC (5 mM, 1 hr) or PEG-CAT (250 U/ml, 1 hr), then challenged with PM at 100 μg/ml. Changes in TER were measured with the ECIS 6 hr post-PM treatment. *p < 0.05 compared to PM only group.

We assessed human lung microvascular EC barrier function as measured by transendothelial electrical resistance (TER), a highly sensitive measurement of permeability. PM challenge (10–100 μg/ml) induced dose- and time-dependent reduction in TER (Additional file 1: Figure S1) or increases in FITC-dextran leakage through EC monolayer (Additional file 1: Figure S2) [20], indicating a loss of EC barrier integrity. At the same time, PM induced a time-dependent (1–6 hr) reduction of the levels of tight junction proteins ZO-1 and ZO-2, but did not affect levels of adhesion junction proteins VE-cadherin or β-catenin (Figure 1A). No obvious cytotoxicity in the ECs was found after PM challenge (100 μg/ml, 0–16 hr) with MTT assay [13] or LDH release assay (Additional file 1: Figure S3).

### PM induces ROS-dependent EC barrier disruption and ZO-1 degradation

PM (100 μg/ml) induced substantial time-dependent ROS production in microvascular ECs as measured by DCFDA oxidation, which peaked around 30–60 min (Additional file 1: Figure S4). EC pretreatment with N-acetyl-cysteine (NAC, 5 mM, 1 hr), an ROS scavenger, or PEG-catalase (PEG-CAT, 250 U/ml, 1 hr), which degrades H_2_O_2_, prevented PM-induced DCFDA oxidation by ROS (Additional file 1: Figure S4). PM produced a sustained time-dependent decrease in TER, with a maximal effect observed at 100 μg/ml (80% decrease in TER), which is similar to its effects on other endothelial cell types, as we have reported previously [13]. NAC pretreatment (5 mM, 1 hr pretreatment) prevented PM-induced ZO-1 degradation (Figure 1B), while NAC (5 mM, 1–24 hr) does not change ZO-1 protein levels by itself (Additional file 1: Figure S5). After ECs were treated with PM (100 μg/ml, 6 hr), ZO-1 was relocated from the cell periphery and degraded, which was followed by gap formation between ECs. VE-cadherin, on the other hand, underwent no relocation or degradation (Figure 1C). NAC pretreatment (5 mM, 1 hr pretreatment) prevented PM-induced ZO-1 relocation and gap formation (Figure 1C). These data strongly indicate that PM causes EC barrier disruption selectively via oxidative tight junction protein degradation. NAC (5 mM) or PEG-CAT (250 U/ml) pretreatment significantly inhibited PM-induced EC barrier disruption as measured by TER (Figure 1D). We also examined the effects of another ROS scavenger EUK-134 on PM-challenged ECs. As NAC, EUK (5 μM, 1 hr pretreatment) attenuated PM-induced ZO-1 degradation and TER reduction (Additional file 1: Figure S6). These results further confirmed that PM induces ROS-dependent EC barrier disruption.

### Activated calpain is required for PM-mediated ZO-1 degradation and EC barrier disruption

**Figure 2:**
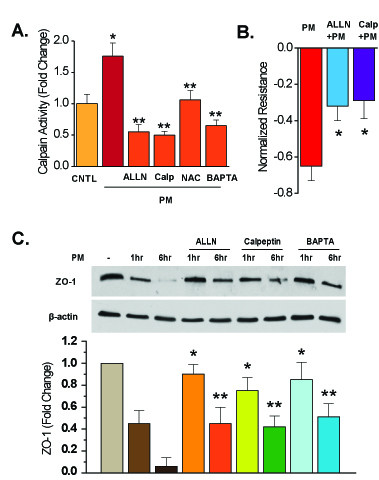
**Activated calpain is required for PM-mediated ZO-1 degradation and EC barrier disruption. (A)** Human lung microvascular ECs grown in 60-mm dishes to approximately 95% confluence were treated with ALLN (30 μM), calpeptin (CALP, 10 μM), NAC (5 mM), or BAPTA-AM (50 μM) for 1 hr, and then challenged with PM (100 μg/ml) for 1 hr. Cell lysates were subjected to calpain activity assay by using the calpain activity kit (Calbiochem). Calpain activity was normalized to protein concentration and expressed as fold changed compared to control. *p < 0.05 compared to control. **p < 0.05 compared to PM only group. **(B)** ECs grown on ECIS gold electrodes were treated with ALLN (30 μM), calpeptin (CALP, 10 μM) for 1 hr, and then challenged with PM (100 μg/ml). Changes in TER were measured with ECIS. *p < 0.05 compared to PM only group. **(C)** ECs grown on 6-well plates were treated with ALLN (30 μM), calpeptin (CALP, 10 μM), or BAPTA-AM (50 μM) for 1 hr, and challenged with PM (100 μg/ml) for 1–6 hr. Cell lysates were analyzed by Western blotting with antibody to ZO-1. Changes in levels of ZO-1 are expressed as fold changes and normalized to β-actin. Shown are representative blots from three independent experiments. *p < 0.05 compared to PM-1 hr group. **p < 0.05 compared to PM-6 hr group

We previously demonstrated that high levels of ROS in endothelial cells activate calpain, a calcium-dependent protease [21]. We therefore investigated the role of calpain in PM-mediated ZO-1 degradation. PM (100 μM, 1 hr) induced a significant increase in calpain activity in ECs, which was inhibited by selective calpain inhibitors ALLN (30 μM, 1 hr pretreatment) and calpeptin (10 μM, 1 hr pretreatment) (Figure 2A). PM-induced calpain activation was also inhibited by BAPTA-AM (50 μM, 1 hr pretreatment), a calcium chelator, and by NAC (5 mM, 1 hr pretreatment) (Figure 2A). We next determined the role of calpain in PM-induced EC barrier disruption. PM-induced reduction in TER was partially inhibited by calpeptin or ALLN (Figure 2B). In parallel, ALLN and calpeptin also significantly prevented PM-induced ZO-1 degradation, as did chelation of intracellular calcium via BAPTA-AM (Figure 2C).

### PM induces calcium leakage via ROS-activated TRPM2

**Figure 3:**
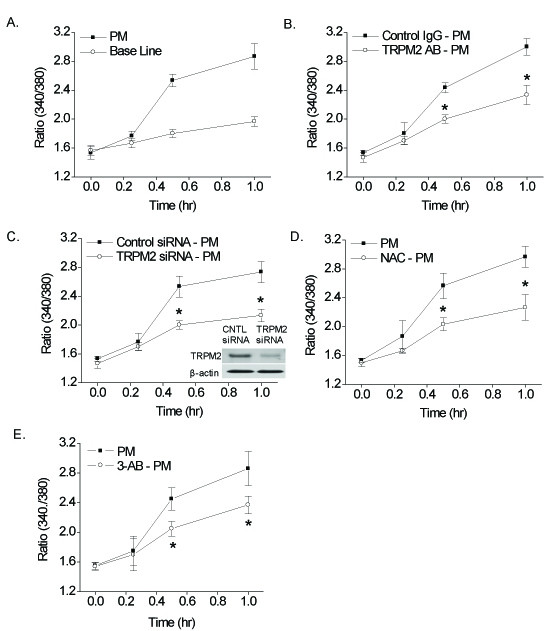
**PM induces calcium leakage via ROS-activated TRPM2. (A)** Human lung microvascular ECs were plated on glass cover slips and loaded with 5 μM Fura-2 AM in 1 ml of basic medium. ECs were rinsed twice and treated with PM (100 μg/ml). Fura-2 fluorescence was measured at excitation wavelengths of 340 and 380 nm and an emission wavelength of 510 nm (0–60 min). Fura-2 has excitation wavelengths of 380 nm in its free form and 340 nm when it is bound to calcium; therefore, relative calcium concentrations are represented by the ratio of 340/380. **(B)** ECs plated on glass cover slips were treated with TRPM2 antibody or control IgG (5 μg/ml, 4 hr pretreatment). ECs were then rinsed twice and subjected to the same calcium measurement procedures with PM treatment (100 μg/ml, 0–60 min). *p < 0.05 compared to control IgG group at the same time point. **(C)** ECs were transfected with TRPM2 siRNA or control siRNA for 48 hrs. ECs were then rinsed twice and subjected to the same calcium measurement procedures with PM treatment (100 μg/ml, 0–60 min). EC cell lysates were analyzed by Western blot with antibodies to TRPM2 and β-actin to confirm silencing. *p < 0.05 compared to control siRNA group at the same time point. **(D-E)** ECs plated on glass cover slips (95% confluent) were treated with NAC (5 mM, 1 hr pretreatment) or 3-AB (1 mM, 1 hr pretreatment). ECs were then rinsed twice and subjected to the same calcium measurement procedures with PM treatment (100 μg/ml, 0–60 min). *p < 0.05 compared to PM-only group at the same time point.

Addition of PM (100 μg/ml) to human lung microvascular ECs produced slow time-dependent Ca^2+^ influx (Figure 3A; Ca^2+^ influx is represented by an increase in the 340/380 ratio of Fura-2 AM.). Notably, this finding of increased intracellular Ca^2+^ is in accordance with our previous finding of increased activated calpain. Under oxidative stress, a key member of the transient receptor potential (TRP) cation channel, member M2 (TRPM2), is activated and causes slow calcium influx [22]. We therefore examined the role of activated TRPM2 in PM-stimulated calcium influx. Anti-TRPM2 blocking antibody (5 μg/ml, 4 hr pretreatment) significantly prevented the PM-induced Ca^2+^ transients compared to ECs treated with control IgG (Figure 3B). Reductions in TRPM2 protein expression (by siRNA) also inhibited the PM-induced Ca^2+^ influx compared to ECs treated with control siRNA (Figure 3C).

We next investigated whether TRPM2 activation was ROS-dependent. Depletion of PM-induced ROS by NAC (5 mM, 1 hr pretreatment) significantly prevented PM-mediated calcium influx (Figure 3D). Under oxidative stress, Poly ADP ribose polymerase (PARP) generates ADP-ribose [23,24], which activates TRPM2 by binding to its carboxyl terminus. The PARP inhibitor 3-aminobenzamide (3-AB, 1 mmol/L, 1 hr pretreatment)significantly reduced PM-induced Ca^2+^ influx (Figure 3E), which further confirmed that TRPM2 is activated by PM via ROS and PARP.

### PM-activated TRPM2 promotes ZO-1 degradation by calpain

**Figure 4:**
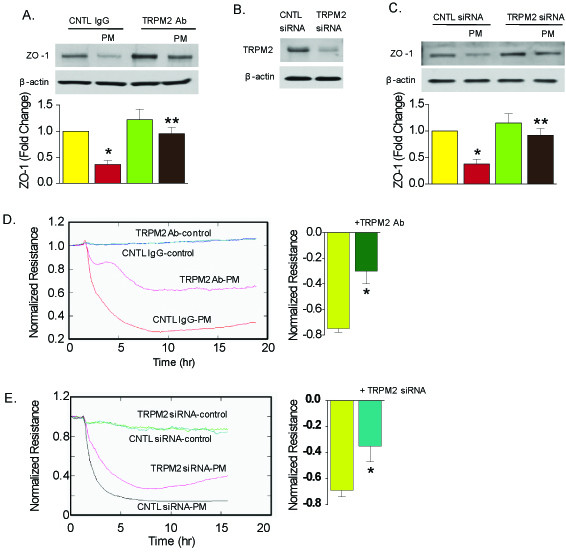
**PM-activated TRPM2 promotes ZO-1 degradation by calpain. (A)** Human lung microvascular ECs grown in 6-well dishes to approximately 95% confluence were treated with TRPM2 antibody or control IgG (5 μg/ml) for 4 hr, and then challenged with PM (100 μg/ml) for 6 hr. Cell lysates were analyzed by Western blotting with ZO-1 antibody. Changes in levels of ZO-1 are expressed as fold changes and normalized to β-actin. Shown are representative blots from three independent experiments. *p < 0.05 compared to control. **p < 0.05 compared to PM challenge. **(B)** ECs grown in 6-well dishes to approximately 80% confluence were treated with TRPM2 siRNA or control siRNA (100 ng/ml) for 48 hr, and cell lysates were analyzed by Western blotting with ZO-1 antibody. **(C)** ECs grown in 6-well dishes to approximately 95% confluence were treated with TRPM2 siRNA or control siRNA (100 ng/ml) for 48 hr, and then challenged with PM (100 μg/ml) for 6 hr. Cell lysates were analyzed by Western blotting with ZO-1 antibody. Changes in levels of ZO-1 are expressed as fold changes and normalized to β-actin. Shown are representative blots from three independent experiments. *p < 0.05 compared to control. **p < 0.05 compared to PM challenge. **(D)** ECs grown on ECIS gold electrodes were treated with TRPM2 antibody or control IgG (5 μg/ml) for 4 hr, and then challenged with PM (100 μg/ml). Changes in TER were measured with ECIS. *p < 0.05 compared to PM-challenged group. **(E)** ECs grown on 100 mm dishes were treated with TRPM2 siRNA or control siRNA (100 ng/ml) for 48 hr, and then plated onto gold electrodes for ECIS measurement. 24 hours after replating, the ECs were challenged with PM (100 μg/ml) and changes in TER were measured with ECIS. *p < 0.05 compared to PM-challenged group.

We next investigated the role of TRPM2 activation in EC barrier function and ZO-1 degradation. TRPM2 neutralizing antibody (5 μg/ml, 4 hr pretreatment) significantly prevented ZO-1 degradation induced by PM (Figure 4A). TRPM2 siRNA (100 ng/ml), which downregulated TRPM2 protein level (Figure 4B), also inhibited PM-induced ZO-1 degradation (Figure 4C). In parallel, antagonizing TRPM2 by either TRPM2 antibody (5 μg/ml, 4 hr pretreatment) or TRPM2 siRNA (100 ng/ml) significantly prevented PM-induced EC barrier disruption (Figure 4d-E), as indicated by TER measurements.

### ROS scavenging or calpain inhibition prevents PM-induced pulmonary inflammation and ZO-1 loss

**Figure 5:**
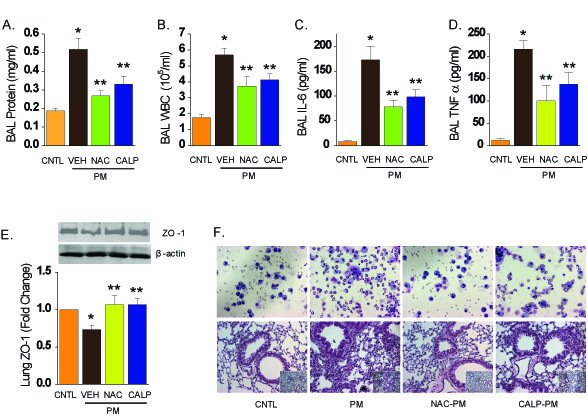
**NAC and calpeptin pretreatment reduces PM-induced pulmonary inflammation and ZO-1 loss in murine lung. **AJ mice (n = 4) were treated NAC (150 mg/kg, intraperitoneal injection) or calpeptin (CALP, 1 mg/kg, intraperitoneal injection) 1 hr before PM challenge (10 mg/kg, intratracheal aspiration). After 24 hr, **(A)** protein concentration of BAL fluids was measured, **(B)** total white blood cell (WBC) in BAL were counted, and **(C-D)** IL-6 and TNF-α levels in BAL were measured. **(E)** Protein was extracted from the mouse lungs and analyzed by Western blotting using ZO-1 and β-actin antibodies. Changes in levels of ZO-1 are expressed as fold changes and normalized to β-actin. *p < 0.05 compared to control. **p < 0.05 compared to PM-challenge. **(F)** BAL cellularity were examined by cytospin slides, and lung tissues were examined by hematoxylin and eosin (H&E) staining.

A PM-mediated murine model of pulmonary inflammation has been well established [12]. We investigated the role of ROS and calpain in PM-induced pulmonary inflammation by examining protein leakage, white blood cell infiltration (inflammatory leukocytes), and the release of proinflammatory cytokines into BAL fluids (Figure 5). Pre-administration of NAC (150 mg/kg) or calpeptin (1 mg/kg) 1 hr before PM challenge (10 mg/kg, 24 hr) significantly attenuated PM-induced protein leakage into BAL fluids (~ 50% reduction, Figure 5A). PM challenge (10 mg/kg, 24 hr) resulted in an increase in inflammatory leukocytes, e.g. neutrophils and eosinophils [12,15], in BAL fluids (Figure 5B). Pre-administration of NAC (150 mg/kg) or calpeptin (1 mg/kg) attenuated PM-induced inflammatory leukocyte infiltration. Furthermore, NAC (150 mg/kg) or calpeptin (1 mg/kg) attenuated the release of PM-induced proinflammatory cytokines IL-6 and THF-α into BAL (~50% inhibition, Figure 5C-D). These results suggest that ROS scavenging by NAC or calpain activity inhibition by calpeptin leads to multiple protective effects including enhancement of lung endothelial barrier function, reduction of inflammatory cell infiltration, and prevention of proinflammatory cytokine release in the lungs of PM-challenged mice. We next examined tight junction ZO-1 levels in the PM-exposed lung. PM challenge (10 mg/kg, 24 hr) induced a significant reduction of ZO-1 protein levels in the murine lung, while pre-administration of NAC (150 mg/kg) or calpeptin (1 mg/kg) 1 hr before PM challenge attenuated PM-induced ZO-1 loss from lung tissues. Taken together, these data suggest a crucial role for the ROS-calpain-ZO-1 signaling pathway in the regulation of EC barrier disregulation in PM-mediated pulmonary inflammation.

### Over-expression of endothelial ZO-1 attenuates PM mediated endothelial barrier disruption *in vitro* and pulmonary inflammation *in vivo*

**Figure 6:**
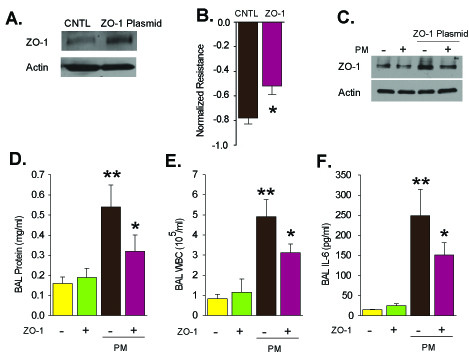
**Over-expression of endothelial ZO-1 attenuates PM-induced EC barrier disruption *in vitro *and pulmonary inflammation *in vivo*. (A)** Human lung microvascular EC were grown to 60% confluence and treated with ZO-1 expression plasmid with X-fect reagent for 48 hr, and over-expression of ZO-1 protein was confirmed by Western blot. **(B)** The ECs were then challenged with PM (100 μg/ml), and changes in TER after 6 hr were measured with ECIS. *p < 0.05 compared to PM-challenged group. AJ mice were treated with ZO-1 expression plasmid with an ACE antibody-conjugated liposome delivery system (5 mg/kg) for 3 days, then challenged with PM (10 mg/kg). After 24 hr of PM exposure, **(C)** lung ZO-1 levels were analyzed with Western blot. Shown is one of the three repeated blots. BAL was collected and **(D)** protein content, **(E)** total white blood cell, and **(F)** IL-6 levels were measured. N = 4. *p < 0.05 compared to PM-challenged group. **p < 0.05 compared to control.

To further confirm the critical role of ZO-1 degradation in EC barrier disruption *in vitro* and pulmonary inflammation *in vivo*, we next examined the beneficial effects of over-expressing ZO-1 protein in endothelial cells *in vitro* and *in vivo*. ZO-1 over-expression (Figure 6A) significantly (but not completely) attenuated PM-induced EC barrier disruption (Figure 6B). These facts demonstrate that endothelial ZO-1 loss contributes to PM-mediated EC barrier disruption. We next over-expressed ZO-1 *in vivo* by a liposome delivery system labeled with ACE antibody, which successfully over-expressed ZO-1 in murine lung tissues (Figure 6C). ZO-1 over-expression significantly attenuated BAL protein leakage (Figure 6D), BAL white blood cell infiltration (Figure 6E), and the release of proinflammatory cytokine IL-6 into BAL (Figure 6F), indicating the crucial role of ZO-1 loss in mediating PM-induced pulmonary inflammation and lung vascular hyperpermeability.

## Discussion

The most significant finding of the present study is the novel characterization of a ROS-dependent pathway that causes calpain-dependent endothelial ZO-1 degradation in response to PM. These data represent the first evidence that calpain signaling, via calcium leakage from activated TRPM2 by ROS, plays a critical role in modulating endothelial cell barrier function, resulting in tight junction protein ZO-1 degradation (Additional file 1 Figure S7). The consequence of ZO-1 degradation is sustained endothelial hyperpermeability and persistent lung inflammation, both of which contribute to variety of acute or chronic cardiovascular disorders [25,26]. These effects were also observed with other types of PM samples (1648a from National Institute of Standards and Technology, fine PM collected from New York city or Baltimore, data not shown), indicating a selective pathogenesis pathway by PM pollution.

Previous studies report that PM triggers the generation of reactive oxygen species or ROS mainly from dysfunctional mitochondria [27,28], and we also noticed the massive generation of ROS by this PM sample is also mainly from mitochondria (unpublished observation). The high iron level of this particular PM might also contribute to the ROS generated via Fenton reactions. ROS released endogenously, have been implicated in the pathophysiology of several lung diseases, including asthma and COPD, as the biochemical mechanisms underlying the urban PM-induced airway inflammation and toxicity [29]. ROS are highly reactive and cause deleterious gene, protein, and tissue effects. ROS are increased in BAL or exhaled breath condensate from patients with inflammatory lung injuries and from people with cardiopulmonary disease who have been exposed to PM [30,31]. This response may reflect the high oxidative potential of fine and ultrafine particulates. Residual oil fly ash (ROFA) and PM1.7-3.5 cause pulmonary inflammation mediated by oxidative stress [32,33]. *In vivo*, exposing rats to PM leads to the formation of free radicals in the lung [34]. Since cardiovascular disease is considered a risk factor of PM-related mortality and morbidity, it is interesting to note that spontaneously hypertensive rats, when exposed to PM, were more susceptible to pulmonary (inflammatory injury) and cardiovascular complications (acute depression of ECG activity) in an oxidant-dependent manner [35]. Besides ROS, PM might trigger adverse outcomes via other potential mechanisms including nonselective phosphatase inhibition (by vanadium) or competitive ion channel inhibition (by nickel) due to the complex and variable chemical components.

In this study, we first define a novel pathway that mediates ROS-dependent tight junction disruption upon particulate matter challenge. Tight junctions, or zonula occludens, are the most apical component of the intercellular junctional complex, which also includes adherens junctions, desmosomes, and gap junctions [36]. ZO-1 was the first tight junction protein to be identified, and ZO-2 and ZO-3 were later isolated as proteins that co-immunoprecipitated with ZO-1 [37,38]. ZO-1 is a peripheral membrane-associated component of the cytoplasmic plaque of tight junctions and is found ubiquitously within tight junctions of epithelial and endothelial cells [39]. ZO-1 interacts with many cellular proteins via its multiple protein-binding domains. ZO-1 has been reported to interact with other ZO family members or claudins via the PDZ domains [40,41]. ZO-1 interacts with the C-terminus of occludin with its GuK domain and the acidic domain [42]. The proline-rich C-terminus of ZO-1 mediates its binding to F-actin *in vitro*, and thus links it to the cytoskeleton [43]. Clearly, ZO-1 interacts with a wide variety of cell skeleton components and plays a central role in orchestrating tight junction complexes. Any dysregulation of ZO-1 in endothelial cells by extracellular stimuli, such as virus shell proteins or alcohol, leads to persistent tight junction disruption and vascular hyperpermeability.

Calpain is a regulator of endothelial integrity which helps control fundamental cellular processes including cytoskeletal remodeling, membrane fusion, cell proliferation and differentiation, and activation of proteolytical cascades leading to apoptosis [44,45]. Under oxidative stress, activated calpain cleaves eNOS and cytoskeletal proteins and induces apoptosis [21,46-48]. Particulate matter induces endothelial cell intracellular oxidative stress, which leads to the activation of calpain, one of the major cytoskeletal regulators. Here we describe the cleavage of tight junction protein ZO-1 by activated calpain both *in vitro* and *in vivo*, indicating that calpain plays a central role in PM-induced endothelial barrier disruption and vascular hyperpermeability. In addition, as activated calpain cleaves other critical cytoskeletal proteins including ezrin and MARCKS protein, the contribution of the other cytoskeletal proteins to the EC hyperpermeability induced by PM needs to be further investigated.

Oxidative calcium influx is mediated by plasma membrane cation-permeable ion channels. The transient receptor potential protein (TRP) and its homologs are cation channels with a tetramer secondary structure which senses diverse stimuli from the extracellular and intracellular environments [49]. Mammalian TRPs comprise six major subfamilies. TRPM2, a member of the TRP channel M2 subtype, is a calcium-permeable channel activated by intracellular messengers such as ADP-ribose [50]. Massive ROS burden induced by PM contributes to DNA oxidation and damage, which activates poly-ADP ribose polymerase (PARP) to initiate DNA repair mechanisms. PARP binds to single-stranded and double-stranded DNA breaks and catalyses the breakdown of NAD into nicotinamide and ADP-ribose, the intracellular agonist of TRPM2 [22,51,52]. Oxidative stress-mediated activation of the PARP pathway serves as the major source of free ADP-ribose production in endothelial cells [53]. Intracellular ADP-ribose activates TRPM2, allowing calcium ions to enter the cell, which in turn trigger numerous physiological and pathological processes.

An important limitation of our study is the high dose of PM that we employed. With 10–30 μg/m^3^ ambient PM level in the US or Europe, it is hardly to achieve a high level of acute PM exposure. While 100 μg/ml (*in vitro*) or 10 mg/kg (*in vivo*) are typical doses used in particulate matter toxicology studies [12,13,27,54-56]. With an assumed ambient PM level of 20 μg/m^3^, one man with 70 kg body weight and 8 m^3^/minute respiration rate would receive a dose of 10 mg/kg corresponding to about 16 years of exposure with 50% deposition rate. As noted, a lot of cities in the developing countries still have high levels of ambient PM. A report by world bank [57] stated that in the year of 2006, extremely high PM10 levels still existed in a lot of cities (μg/m^3^): Nyala in Sudan (359), Kano in Nigeria (283), Hyderabad in Pakistan (239), Maroua in Cameroon (228), Muzaffarpur in India (218), N'DJAMENA in Chad (204), Segou in Mali (200), Erbil in Iraq (195), Shubra-El-Khema in Egypt (186), DHAKA in Bangladesh (174). Over 13 million people live with more than 200 μg/m^3^ ambient PM in Pakistan, which results over 20 mg inhaled per week, or 16 mg/kg per year.

Extensive epidemiologic and experimental evidence has demonstrated that particulate air pollution directly causes cardiopulmonary damage. Our observations demonstrate a novel mechanism of PM-mediated disruption of endothelial barrier function which is attributable to ZO-1 degradation by calpain, which is activated by extracellular calcium leakage through oxidant-sensitive TRPM2 channels. Therefore, inhibition of ROS/TRPM2/calpain/ZO-1 degradation may provide useful therapeutic strategies for the treatment of endothelial barrier dysfunction and lung inflammation.

## Competing interests

The authors declare that they have no competing interests.

## Authors’ contributions

TW designed & performed research, analyzed & interpreted data, and wrote the manuscript. LW, LM, GDL, JHS, BM, and PVU performed research and analyzed data. JMS, ASG, and PNB provided PM sample and reviewed the manuscript. VN interpreted data and reviewed the manuscript. JGNG designed research, interpreted data, and wrote the manuscript. All authors read and approved the final manuscript.

## Disclaimer

Although the research described in this article has been funded in part by the United States Environmental Protection Agency through grant/cooperative agreement #RD-83241701, it has not been subjected to the Agency’s required peer and policy review and therefore does not necessarily reflect the views of the Agency and no official endorsement should be inferred.

## Disclosures

None.

## Supplementary Material

Additional file 1 **Figure S1.** (A-B) PM induces dose-dependent reduction in transendothelial resistance (TER). (C) PM induces dose-dependent (6 hr) reduction of ZO-1 protein levels. **Figure S2.** PM induced FITC-dextran leakage across EC monolayer. **Figure S3.** PM (100 μg/ml, 1-16 hr) does not induce LDH release from human ECs. **Figure S4.** NAC or PEG-CAT attenuates PM-induced ROS in ECs. **Figure S5.** NAC (5 mM, 1-24 hr) does not change ZO-1 protein levels in human ECs. **Figure S6.** EUK-134 (5 μM, 1 hr pre-treatment) attenuates PM (100 μg/ml, 6 hr)-induced ZO-1 degradation and TER reduction. **Figure S7.** We hypothesize that PM induces EC barrier disruption in delayed phase (via ZO-1 degradation) and acute phase (via stress fiber formation). Click here for file
