# Supplementary material for: Particulate matter air pollution disrupts endothelial cell barrier via calpain-mediated tight junction protein degradation
Source: Part Fibre Toxicol. 2012 Aug 29;9:35. doi: 10.1186/1743-8977-9-35 (PMC3489700; doi:10.1186/1743-8977-9-35)
Supplement: Additional file 1 — Figure S1. (A-B) PM induces dose-dependent reduction in transendothelial resistance (TER). (C) PM induces dose-dependent (6 hr) reduction of ZO-1 protein levels. Figure S2. PM induced FITC-dextran leakage across EC monolayer. Figure S3. PM (100 μg/ml, 1-16 hr) does not induce LDH release from human ECs. Figure S4. NAC or PEG-CAT attenuates PM-induced ROS in ECs. Figure S5. NAC (5 mM, 1-24 hr) does not change ZO-1 protein levels in human ECs. Figure S6. EUK-134 (5 μM, 1 hr pre-treatment) attenuates PM (100 μg/ml, 6 hr)-induced ZO-1 degradation and TER reduction. Figure S7. We hypothesize that PM induces EC barrier disruption in delayed phase (via ZO-1 degradation) and acute phase (via stress fiber formation). [file 1743-8977-9-35-S1.doc]

# *Online Supplement Material for Part Fibre Toxicol.*

# Particulate Matter Air Pollution Disrupts Endothelial Cell Barrier via Calpain-Mediated Tight Junction Protein Degradation

Ting Wang^1^, Lichun Wang^1^, Liliana Moreno-Vinasco^1^, Gabriel D. Lang^1^, Jessica H. Siegler^1^, Biji Mathew^1^, Peter V. Usatyuk^1^, Jonathan M. Samet^2^, Alison S. Geyh^3^, Patrick N. Breysse^3^**, Viswanathan** Natarajan^1^, Joe G. N. Garcia^1,4^

^1^Institute for Personalized Respiratory Medicine, Section of Pulmonary, Critical Care, Sleep and Allergy, Department of Medicine, University of Illinois at Chicago, Chicago, IL

^2^Department of Preventive Medicine, Keck School of Medicine, University of Southern California, Los Angeles, CA

^3^Department of Environmental Health Science, Bloomberg School of Public Health, Johns Hopkins University, Baltimore, MD

^4^Correspondence to

Joe GN Garcia, MD

Institute for Personalized Respiratory Medicine

University of Illinois at Chicago

1737 W Polk Street (MC 672)

Chicago IL 60612


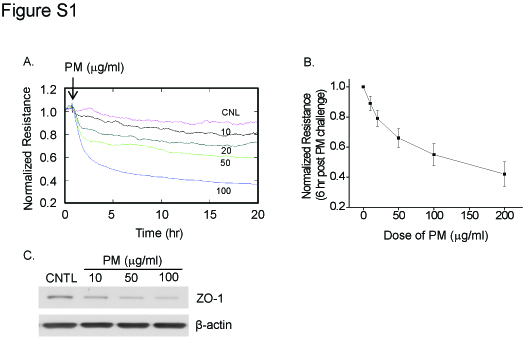


Figure S1. (A-B) PM induces dose-dependent reduction in transendothelial resistance (TER). (C) PM induces dose-dependent (6 hr) reduction of ZO-1 protein levels.


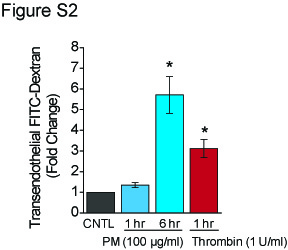


Figure S2. PM induced FITC-dextran leakage across EC monolayer.


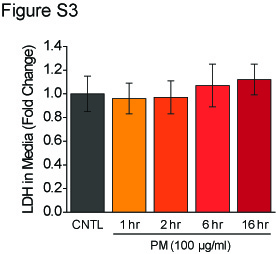


Figure S3. PM (100 µg/ml, 1-16 hr) does not induce LDH release from human ECs.


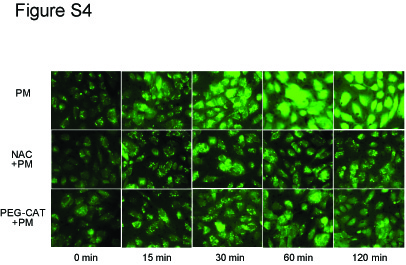


Figure S4. NAC or PEG-CAT attenuates PM-induced ROS in ECs.


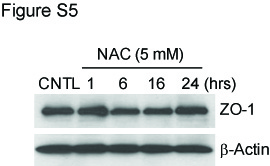


Figure S5. NAC (5 mM, 1-24 hr) does not change ZO-1 protein levels in human ECs.


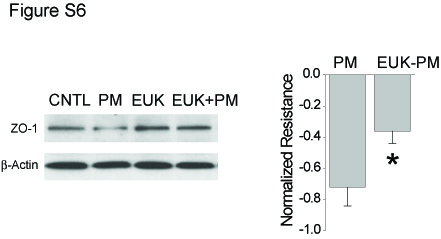


Figure S6. EUK-134 (5 µM, 1 hr pre-treatment) attenuates PM (100 µg/ml, 6 hr)-induced ZO-1 degradation and TER reduction.


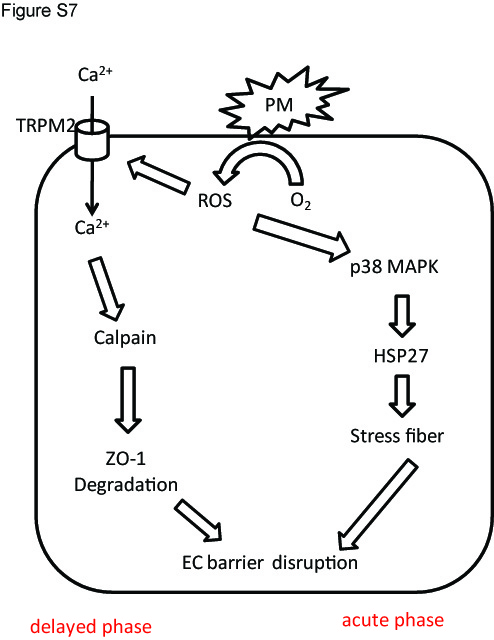


Figure S7. We hypothesize that PM induces EC barrier disruption in delayed phase (via ZO-1 degradation) and acute phase (via stress fiber formation).
